# A sequence-based method to predict the impact of regulatory variants using random forest

**DOI:** 10.1186/s12918-017-0389-1

**Published:** 2017-03-14

**Authors:** Qiao Liu, Mingxin Gan, Rui Jiang

**Affiliations:** 1 MOE Key Laboratory of Bioinformatics; Bioinformatics Division and Center for Synthetic and Systems Biology, TNLIST; Department of Automation, Tsinghua University, Beijing, 100084 China; 2 Department of Management Science and Engineering, Dongling School of Economics and Management, University of Science and Technology Beijing, Beijing, 100083 China

## Abstract

**Background:**

Most disease-associated variants identified by genome-wide association studies (GWAS) exist in noncoding regions. In spite of the common agreement that such variants may disrupt biological functions of their hosting regulatory elements, it remains a great challenge to characterize the risk of a genetic variant within the implicated genome sequence. Therefore, it is essential to develop an effective computational model that is not only capable of predicting the potential risk of a genetic variant but also valid in interpreting how the function of the genome is affected with the occurrence of the variant.

**Results:**

We developed a method named *kmer*Forest that used a random forest classifier with *k*-mer counts to predict accessible chromatin regions purely based on DNA sequences. We demonstrated that our method outperforms existing methods in distinguishing known accessible chromatin regions from random genomic sequences. Furthermore, the performance of our method can further be improved with the incorporation of sequence conservation features. Based on this model, we assessed importance of the *k*-mer features by a series of permutation experiments, and we characterized the risk of a single nucleotide polymorphism (SNP) on the function of the genome using the difference between the importance of the *k*-mer features affected by the occurrence of the SNP. We conducted a series of experiments and showed that our model can well discriminate between pathogenic and normal SNPs. Particularly, our model correctly prioritized SNPs that are proved to be enriched for the binding sites of FOXA1 in breast cancer cell lines from previous studies.

**Conclusions:**

We presented a novel method to interpret functional genetic variants purely base on DNA sequences. The proposed *k*-mer based score offers an effective means of measuring the impact of SNPs on the function of the genome, and thus shedding light on the identification of genetic risk factors underlying complex traits and diseases.

## Background

With great efforts in the past decade, genome-wide association studies (GWAS) have discovered a number of potential associations between genetic variants and human inherited diseases or traits [1, 2]. Nevertheless, most of such variants spread over noncoding regions of the entire genome [3, 4], making the interpretation of the identified associations and the final determination of causative variants a great challenge. A common agreement is that the occurrence of a variant may disrupt its hosting regulatory element, result in the loss of function, and hence cause the development of a disease. According to this understanding, the precise prediction of the implication of a variant in a non-coding region is not only crucial to the interpretation of the function of regulatory elements but also urgent to the develop of an effective model for identifying causal variants.

Toward this goal, computational approaches have been proposed to predict functionally damaging effects of genetic variants in the whole genome level [5–7]. For example, CADD [8] and GWAVA [9] integrated a number of genomic and epigenomic annotations to predict functional implications of all possible genetic variants in the human genome under the binary classification framework. Some methods mainly focus on variants occurring in such specific type of regulatory regions as transcription factor (TF) binding sites because it has been revealed that genetic variants occur in transcription factors binding sites can affect cellular phenotype and gene expression [10]. To mention a few, ChroMos, an integrated web-tool for SNPs classification and prioritization with the combination of genetic and epigenetic data [11]. HaploReg, another tool based on quantifying the difference between reference and alternate alleles in genetic context of canonical TF binding motifs [12]. DeepBind [13] identify sequence specificities of DNA- and RNA-binding proteins from experimental data by using the deep learning technology.

From another perspective, chromatin accessibility is one of the basic problem in epigenomics. When DNA molecule fits into the microscopic nucleus which will wrap around special histone protein and package into a fiber known as chromatin, some regions of chromatin will remain accessible to transcription factors (TF) and other cellular machines involved in gene expression while some other regions are unavailable to any cellular machinery. We refer to these two chromatin states as open (accessible) and close (inaccessible), respectively. The open regulatory regions often work together with transcription factors, RNA polymerases and other cellular regulatory machines [14]. Therefore, the chromatin state is a quite important factor to understand the information flow and the regulatory mechanism in the cell. In this sense, the precise prediction of chromatin accessibility has a significant meaning in exploring the function of regulatory variants in genome.

In general, there are two complementary approached to detect putative accessible chromatin regions. The first method is called comparative genomics, which identities relative regions by their sequence conservation across different species, based on the generally accepted hypothesis that functionally important DNA regions are under purifying selection. Naturally, conserved noncoding sequences are candidates for putative open regions. Many relative approaches are already successfully used to detect regulatory regions [15–17]. However, these conservation-based approaches still have some limitations due to the fact that the function and spatio-temporal specificity of conserved noncoding sequences (CNSs) cannot be determined by conservation alone. It is therefore necessary to incorporate additional information. More importantly, some studies have shown that noncoding sequences that lack conservation may still contain functional regulatory elements [18, 19]. The second one is called functional genomics approaches. It is an experimentally driven approach that utilize developed techniques of microarray hybridization or massively sequencing technology. These techniques often combine with chromatin immunoprecipitation on specific transcription factors [20], coactivators [21, 22].

In this paper, we propose a computational approach named *kmer*Forest, a sequence-based method that uses *k*-mer counts as features and a random forest method as the classifier [23]. We adopt the random forest method because it has been successfully applied in many bioinformatics problems, including the gene selection and classification [24], the identification of DNA binding proteins [25, 26] and the detection of causative SNPs [27, 28]. Our *kmer*Forest approach applies the random forest model to capture the sequence elements of chromatin accessibility from the viewpoint of binary classification. With the model obtained, we assess the contribution of a *k*-mer to the classification accuracy by conducting a series of permutation experiments, and we prioritized the *k*-mers according to their contributions. Finally, we use the *k*-mer feature importance to discriminate pathogenic single nucleotide variants and evaluate the impact of single nucleotide polymorphism (SNP). In a series of experiments, our *kmer*Forest method outperforms the existing state-of-the-art approach, kmer-SVM [29], in a variety of cell lines. Based on this model, we introduced a score called the mean decrease accuracy (MDA) to evaluate input features. We then calculated MDA scores for all the pathogenic and normal SNPs. Hopefully, a significant different distribution of two type SNPs was found, revealing the effectiveness of our model. Specifically, our model correctly predicted the impact of a SNP rs4784227 on FOXA1 binding, while Delta-SVM [30] failed.

## Methods

### *k*-mer feature

*k*-mer is a relatively simple sequence feature. It is typically used during the sequence assembling, but it can also be used in the sequence alignment [31]. *k*-mers refers to all possible subsequences of length *k* that are contained in the sequence. As for DNA sequence with length *L*, the total amount of *k*-mers is calculated by *L*-*k* + 1 while each nucleotide position has four possibilities (A, C, G, T). If we encode each nucleotide with 1, 2, 3, 4 for A, C, G and T. we can easily get a feature vector from every DNA sequence with the dimension 4^*k*^, namely ${\overrightarrow{\mathrm{f}}}^{\mathrm{s}}={\left({\mathrm{x}}_1^{\mathrm{s}},{\mathrm{x}}_2^{\mathrm{s}},\dots, {\mathrm{x}}_{\mathrm{n}}^{\mathrm{s}}\right)}^{\mathrm{T}}$. Generally, *k* is set to 4 ~ 10 in the case of DNA sequence. However, the dimension will rise in an exponential speed when *k* rises which means we will come into a curse of dimensionality. For example, the dimension will come to over one million when *k* = 10. Luckily, we can use Jellyfish to fast count the *k*-mer of DNA sequence using parallel computation [32].

### Random forest

Random forest is an ensemble machine learning algorithm for classification and regression which is constructed by a multitude of full depth decision trees without pruning [33]. The output will consider the prediction of each decision tree and make a final decision. In this algorithm, “stochastic discrimination” approach is proposed in the “bagging” idea and random selection of features which means that one sample may be selected more than one time. Such strategies can effectively decrease the risk of overfitting when applied to our problem with large dimension. We use out-of-bag (OOB) data to estimate the mean decrease accuracy (MDA). Random forest has been widely used in the prediction of DNA-binding proteins [25, 26], microarray data classification [24, 34] and many other biology problems.

### Integrate *k*-mer with MSA

We collected the multiple sequence alignment (MSA) data of 100 mammal species from UCSC Genome Browser. We then calculated the frequency noted as *fre* of human’s nucleotide appeared in other species. The original feature vector of a sequence sample ${\overrightarrow{f}}^s={\left({x}_1^s,{x}_2^s,\dots, {x}_n^s\right)}^T$ (*n* = 4^*k*^) then can be rewritten as ${\overrightarrow{f}}^{s^{*}}={\left({x}_1^{s^{*}},{x}_2^{s^{*}},\dots, {x}_n^{s^{*}}\right)}^T$ which

1 $$ {x}_i^{s^{*}}=\frac{1}{k}{\displaystyle \sum_{j=1}^{x_i^s}}{\displaystyle \sum_{q=1}^k}fr{e}_{q,j} $$

Note that ${x}_i^{s^{*}}\le {x}_i^s$ and ${x}_i^{s^{*}}={x}_i^s$ if and only if *fre*_*q*, *j*_ = 1 for all 1 ≤ *q* ≤ *k* and 1 ≤ *j* ≤ *x*_*i*_^*s*^. The new equation (1) not only consider the *k*-mer frequency but also the conservation of related sequences which makes our model more powerful in discriminating chromatin open regions. We should note that the change of the input scalability will not influence the performance much due to the great robustness in the scalability of input data.

### MDA score calculation

One of the most importance steps of our MDA-RF model is to realise the calculation of the MDA score. The concise algorithm of mean decrease accuracy (MDA) calculation can be presented as follows:

As our MDA algorithm contains huge dimension due to the sparse *k*-mer features which will consume much time for computation, our strategy is to store the feature of each sample in the sparse format of libSVM [35] to reduce the memory need. Besides, we used OpenMP library in our program to parallel our algorithm in multithreads as each decision tree is relatively independent. Such strategy can take full advantage of the computing resources which can largely help us accelerate the algorithm.

## Results

### Data sources

We collected 210 DNase-seq datasets across different human cell lines from the ENCODE project [36]. These experiments were carried out across different systems and tissues including normal cell lines and cancer cell lines. Besides, we collected the multiple sequence alignment (MSA) data of 100 mammalians from the UCSC Genome Browser [37] in order to capture the information of sequence conservation. We collected 2977 pathogenic SNPs form the HGMD database [38], 701,984 normal SNPs from the 1000 Genomes project [39] and the associated variants set (AVS) for breast cancer [40].

### Overview of the *kmer*Forest model

**Fig. 1:**
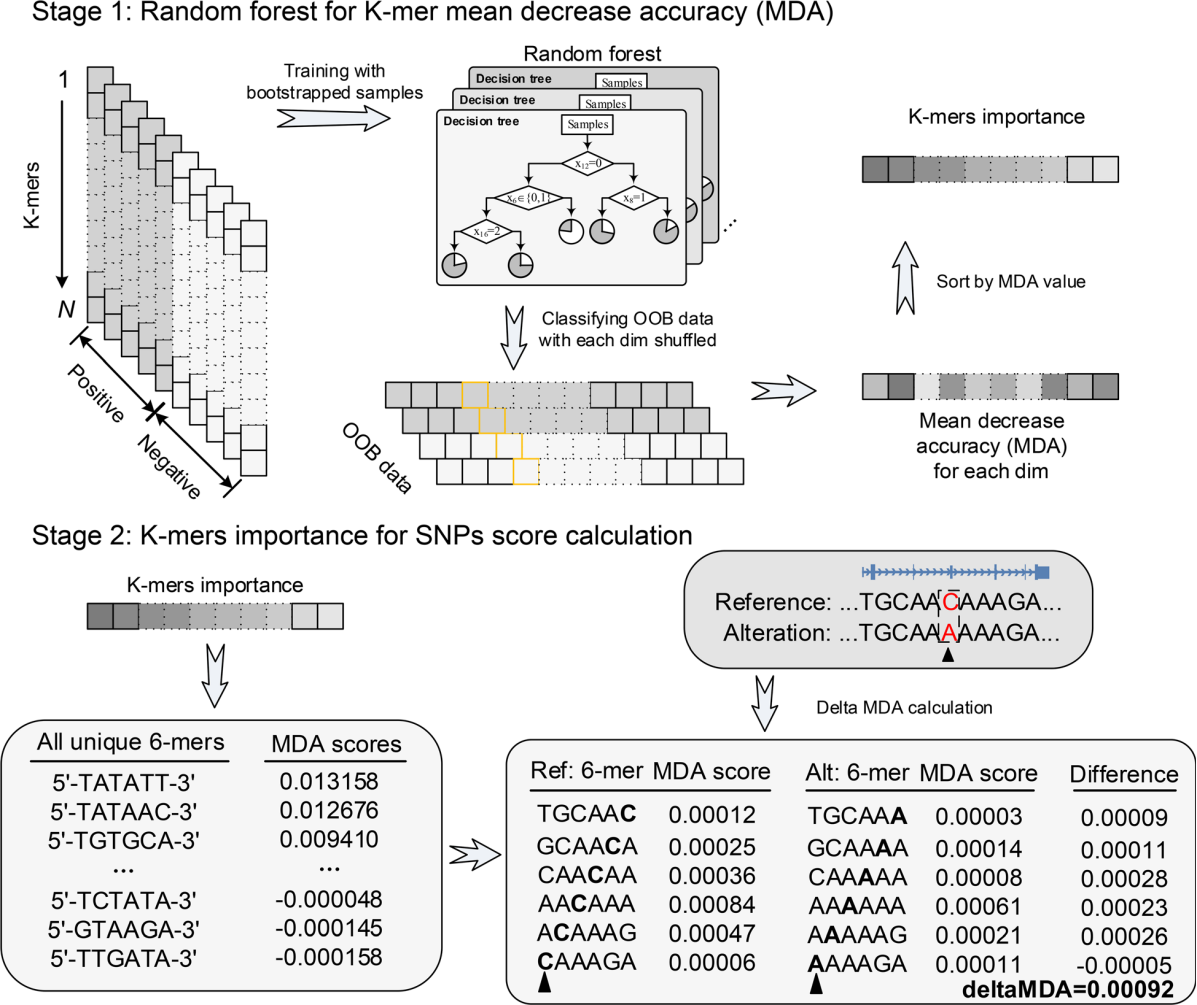
The schematic of *kmer*Forest model. Bootstrapped samples are trained with a random forest. Then out-of-bag samples are classified with the trained model to obtain the MDA score of each *k*-mer. All the MDA score will be sorted as the *k*-mer importance

Our method, named *kmer*Forest, is a machine learning model that targets on discriminating accessible chromatin regions and prioritizes *k*-mer features. As illustrated in Fig. 1. In the first stage, a random forest model is trained with bootstrapped positive samples extracted from DNase-seq data and negative samples obtained from random genomic sequences. Then the trained model classifies out-of-bag (OOB) data with each dimension shuffled to obtain the mean decrease accuracy (MDA) score of each K-mer (see Method). In general, the drop of the mean decrease accuracy (MDA) when shuffling one dimension of the OOB data indicates the importance of the related *k*-mer. Therefore, the quantization model of feature importance is built with MDA score calculation. Then we prioritize all the *k*-mers by sorting their MDA scores, obtaining the *k*-mer importance that will be utilized in evaluating the SNPs in the next stage. In the second stage, we use the calculated MDA scores to evaluate a single nucleotide polymorphism (SNP) by considering the *k*-mers affected by the occurrence of the SNP. Note that a SNP will only cause the alternation of neighboring *k*-mers, and the evaluation of a SNP is the accumulative changed scores of all affected neighboring *k*-mers. In order to comprehensively evaluate the effectiveness of our *kmer*Forest model, we conduct a series of experiments to verify the advantage of our model comparing to existing methods.

### *kmer*Forest outperforms state-of-the-art methods in binary classification

**Fig. 2:**
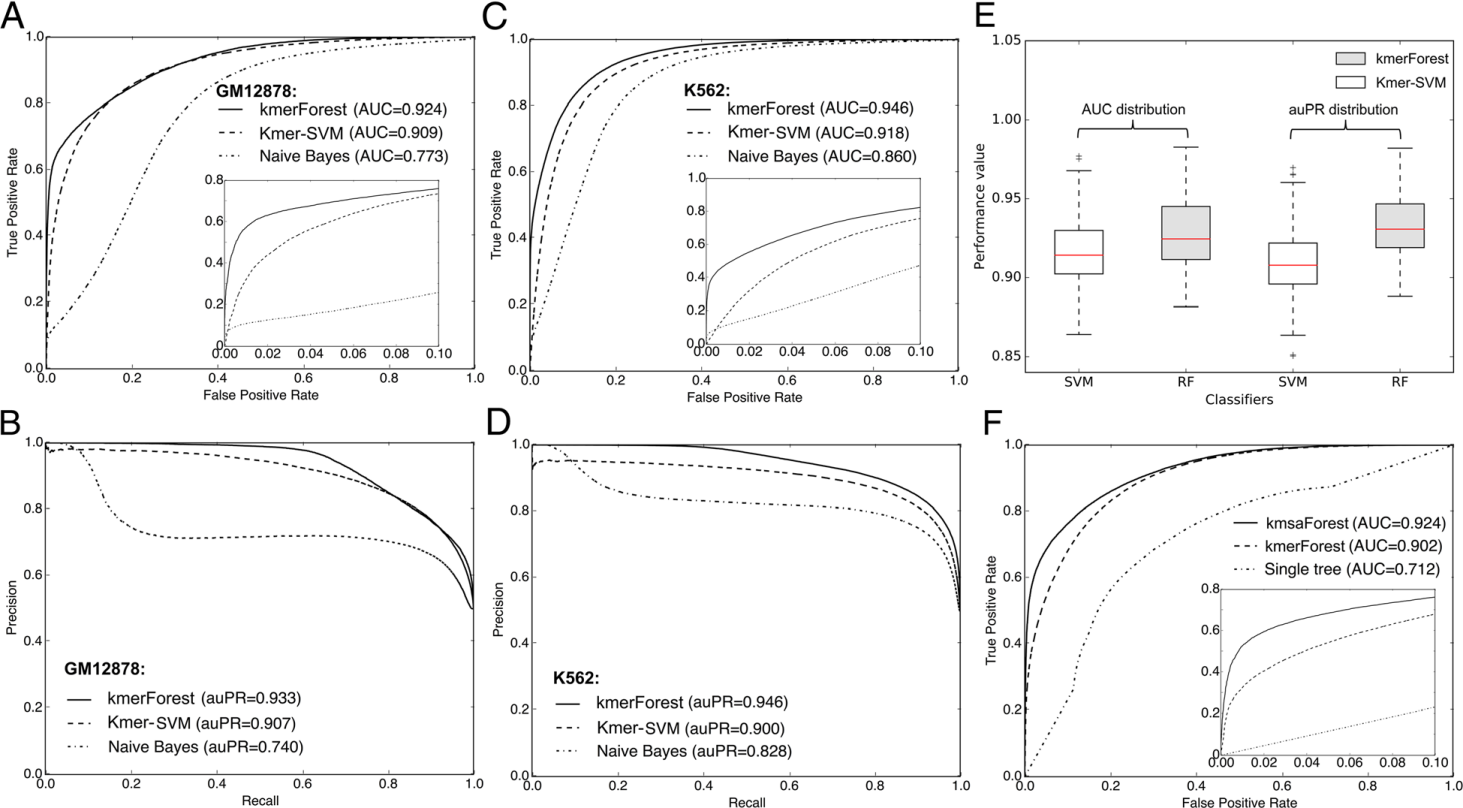
Performance in the binary classification. **a** ROC curve in GM12878 cell line. **b** PR curve in GM12878 cell line. **c** ROC curve in K562 cell line. **d** PR curve in K562 cell line. **e** Distribution of AUC and auPR across 210 cell lines. **f** ROC curve combined with MSA information

We first compared our *kmer*Forest classifier to kmer-SVM [29], considering DNase-seq data from different cell lines in the viewpoint of binary classification. We treated chromatin state as open or close, and we obtained positive samples by extracting putative open regions from peaks of DNase-seq signals. We obtained negative samples by directly sampling random genomic sequences from the entire human genome. When comparing our method to kmer-SVM [29] and a common used Naïve Bayes classifier, *kmer*Forst and Kmer-SVM always achieve better performance than the baseline Naïve Bayes classifier in all experiments. More importantly, *kmer*Forest could achieves higher AUC than Kmer-SVM in 189 out of 210 different cell lines experiments. We listed two representative cell lines, GM12878 and K562 in Fig. 2 (a and c). Our model could achieve a better performance than the other two methods in 90% of the cell lines experiments considering the same input *k*-mer features. Particularly, when we zoom in the ROC curve, we find that our model can achieve a significant higher true positive rate when the false positive rate is relatively small. Fig. 2e shows the distribution of the AUCs and auPRs (area under PR curve) from 210 different cell lines experiments. We observe a significant higher performance of the *kmer*Forest model when compared with the other two models.

### Integration with MSA to improve performance

In order to further improve the performance of the *kmer*Forest classifier, we considered the conservation of the sequences based on the generally accepted hypothesis that the functional regions are under purifying selection. Sequences with strong conservation are the theoretical candidates for putative open regions. As the accessible chromatin regions tend to have a stronger conservation than the close regions. Based on the above, we collected multiple sequence alignment (MSA) data of 100 mammal species from UCSC Genome Browser. We then combined *k*-mer features with the information of sequence conservation from multiple sequence alignment (MSA) and formed a new classifier called *kmsa*Forest (see Methods). The new classify not only consider the k-mer occurrences in sequences but also the conservation information of all the sequences. We designed a series of experiments to see the improvement after integrating with conservation information. In 210 different cell lines experiments, the *kmsa*Forest classifier can effectively improve the AUC by 1 to 2% averagely compared to original *kmer*Forest model. The representative experiment in cell line H1-hESC is showed in Fig. 2f. Note that the performance of single decision tree is also included as a baseline. After integrating the multiple sequence alignment (MSA) information, the new classifier *kmsa*Forest can achieve a better performance which implies that the information of sequence conservation can offer an another perspective of inferring chromatin states.

### *kmer*Forest discriminates pathogenic SNPs from normal ones

**Fig. 3:**
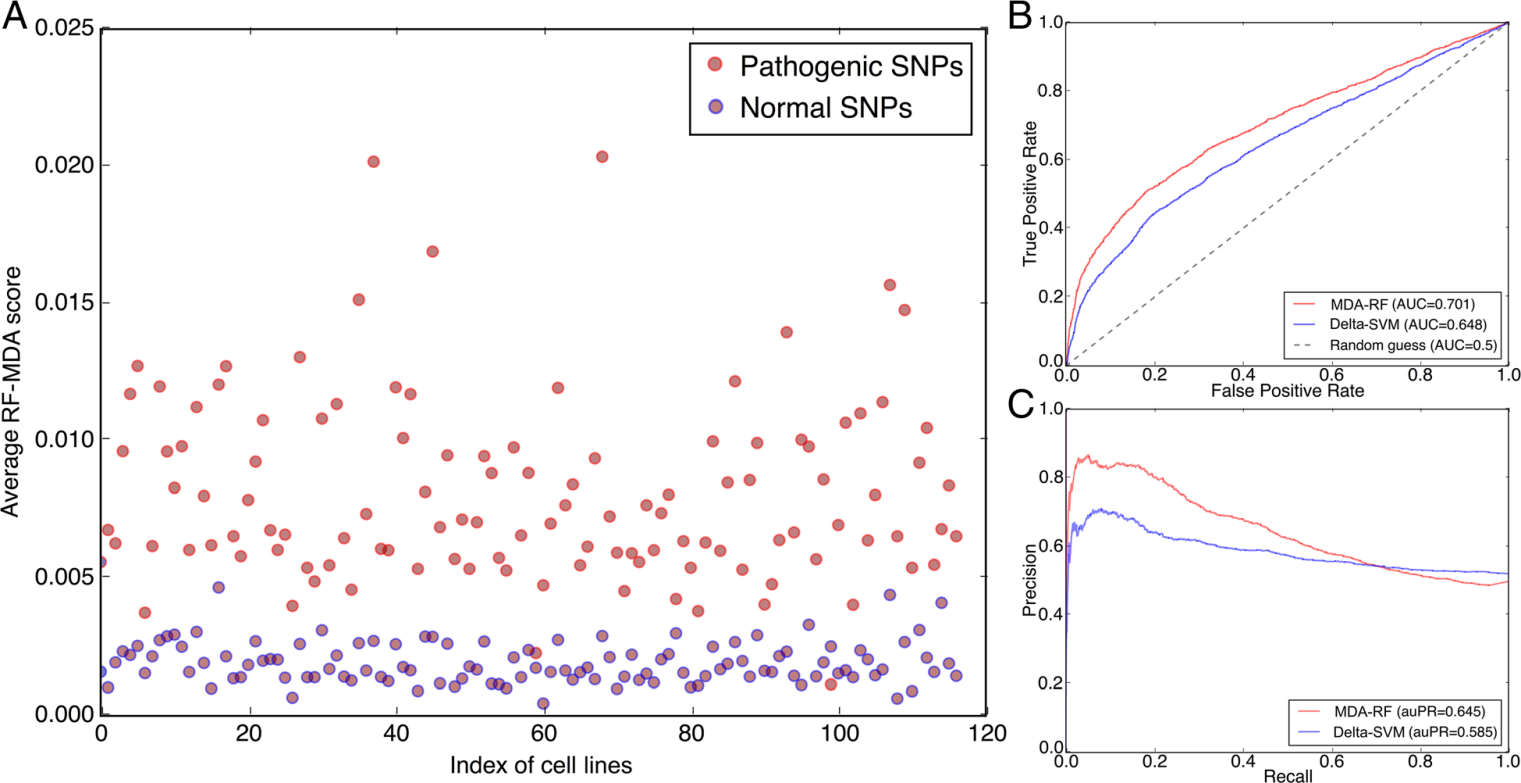
Discriminate two type of SNPs. **a** Distribution of average score in 120 cell lines. **b** ROC curve of SNPs scores. **c** PR curve of SNPs scores

Based on the well-performed random forest classifier, we built a *kmer*Forest model to obtain the importance of all the *k*-mers according to their MDA scores. We then utilized the *k*-mer importance to evaluate the impact of SNPs. We collected 2977 pathogenic SNPs from the HGMD database [38] and 701,984 normal SNPs from the 1000 Genomes project [39]. We then selected at random 3000 SNPs out of these normal SNPs as the negative samples. Note that we selected all the SNPs which are located in putative regulatory regions of human genome, and thus the pathogenic SNPs can be regarded as the disruption of functional regulatory elements. We used our *kmer*Forest model to discriminate the SNPs according to their average MDA score as shown in Fig. 3. As we have collected DNase-seq data across 210 different cell lines in the ENCODE project, we trained our *kmer*Forest model with 210 datasets respectively in order to calculate the average MDA score for all the pathogenic and normal SNPs in each cell line experiment. A significant different distribution of two type of SNPs can be observed in Fig. 3a. The pathogenic SNPs has obviously higher average MDA scores than the normal SNPs, indicating the disruption of regulatory elements when the mutation occurs in functional genomic sequences. In order to compare our model with kmer-SVM [29], we used the SVM weights as the evaluation of each k-mer features after training with the same datasets, and then we calculated the score of each SNP in the same way as *kmer*Forest thus forming the Delta-SVM model. Comparing to our *kmer*Forest model, our method can achieve better performance than Delta-SVM when executing a binary classification on two type of SNPs. The ROC and PR curves are shown in Fig. 3b and c respectively. The AUC value of our model exceeds the Delta-SVM model by about 5%. Such superiority can also be observed in the PR curve. In summary, our *kmer*Forest model can discriminate between two types of SNPs better than Delta-SVM model in evaluating SNPs, revealing that our model could give a more accurate estimation of the impact of SNPs. Our genomic variants evaluation model can further help us predict the impact of possible mutations occur in regulatory regions of genome.

### Application of *kmer*Forest in prioritizing linked-SNPs in breast cancer cell line

**Fig. 4:**
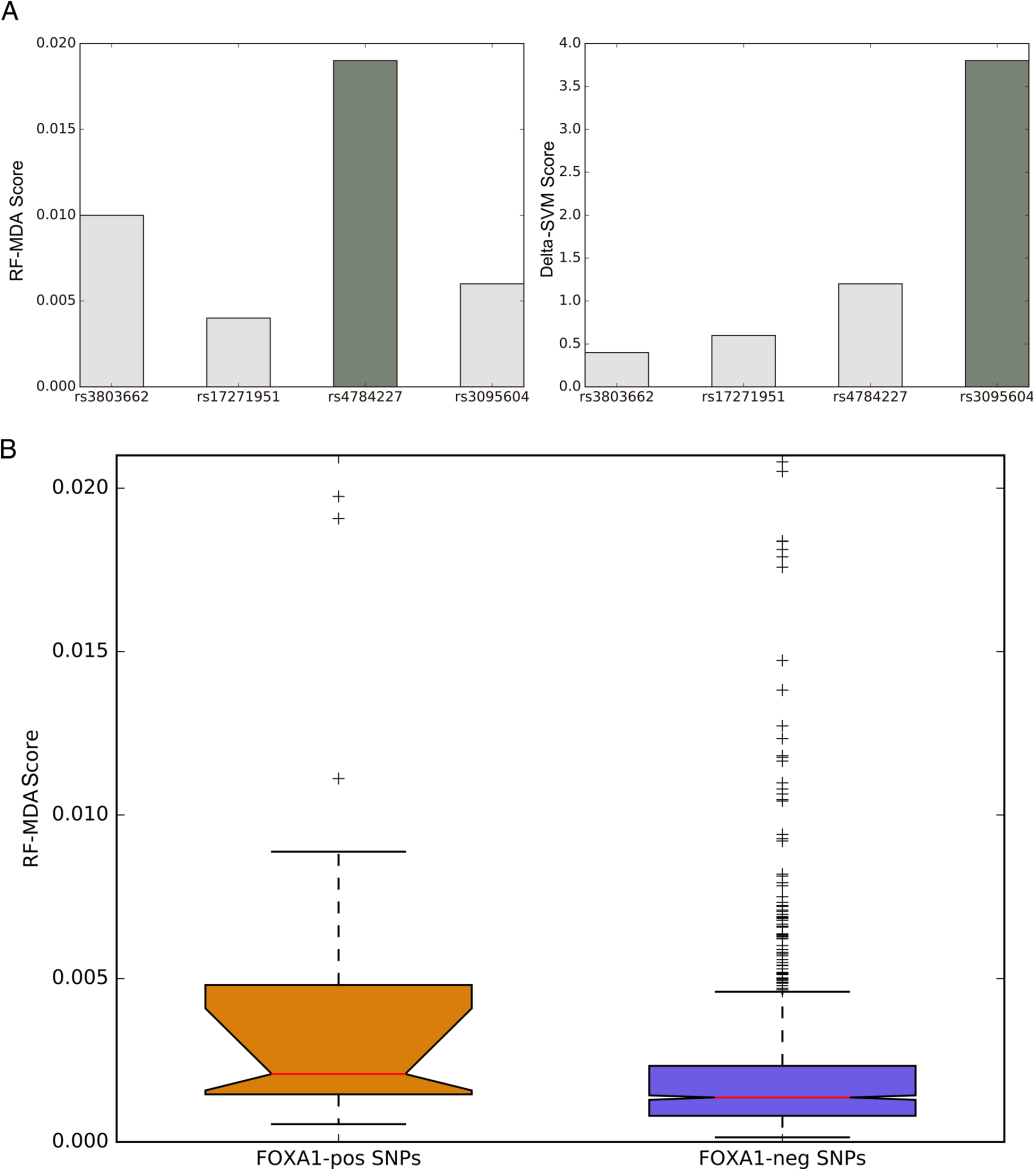
Detection in risk-associated SNPs. **a** The score of risk-associated SNP rs4784227 and other three SNPs in AVS **b** The scores distribution of 44 risk-associated SNPs and 1315 SNPs in AVS

To further demonstrate the application of our *kmer*Forest model, we applied it in post-GWAS analysis. In general, a disease is often associates with more than multiple SNPs in a GWAS. SNPs tend to have an inner association mechanism in the development of the disease. We collected a breast cancer associated variant set (AVS) from a previous study [40]. Briefly, this data set is composed of 44 risk-associated SNPs that were discovered from GWAS and other 1315 “linked” SNPs that were not discovered in GWAS but have strong linkage disequilibrium with the risk-associated SNPs. Previous studies have demonstrated that breast cancer associated SNPs are quite enriched for the binding sites of a transcription factor called FOXA1, which is crucial for chromatin accessibility and nucleosome positioning [41, 42]. Among all the SNPs associated with breast cancer, the rs4784227 is one of the risk-associated SNPs that is believed to disrupt the binding of FOXA1 to accessible chromatin [40, 43]. We trained our *kmer*Forest and Delta-SVM [29] with the same DNase-seq data from breast cancer cell line MCF-7 in the ENCODE project, then we used the two models to evaluate rs4784227 and other linked SNPs collected in AVS (rs3803662, rs17271951, rs3095604) respectively. As is shown in Fig. 4a, our method can correctly predict the impact of rs4784227 while Delta-SVM failed. Next we used our model to evaluate all the SNPs in AVS, the 44 risk-associated SNPs have significant higher MDA scores than the rest of SNPs in AVS (Fig. 4b). According to our MDA scores, we can have an intuitionistic sense of the danger of all the breast cancer associated SNPs.

## Conclusions

In this study, we have proposed a *kmer*Forest model and shown that our method can precisely predict the putative regulatory sequences according to the basic *k*-mer feature without any prior knowledge. Our method outperforms other approaches using only general genomic sequence information. The *kmer*Forest model can effectively help us to discriminate accessible chromatin regions from pure genomic context. Such sequence features identified by our method can be regarded as the functional sequence elements which are the candidates for consensus motifs such as TFBSs. The combined model *kmsa*Forest not only consider the *k*-mer frequency but also the sequence conservation which can always achieve a better performance. This distinction suggests that predictive sequences features may be more evolutionarily conserved. Based on the above, we further developed an application for evaluating the regulatory variants. By prioritizing all the *k*-mers and calculating the MDA scores, we can give an intuitionistic description of the pathogenesis of SNPs associated with diseases. A series of experiments have showed that our method framework can discriminate the pathogenic SNPs from normal SNPs better than the deltaSVM model in previous study. So building such a quantitative model for evaluating the regulatory variants has significant meaning in predicting the impact of special mutation in regulatory genome regions and interpreting the consequence of a variant associated with diseases.

## Discussion

We realize that there are many aspects for us to generalize and improve our model. First, our method is mainly motivated by building the effective model for evaluating regulatory variants associated with diseases. However, considering the accumulative impact of neighboring *k*-mers may ignore the spatial effect of *k*-mers and the interaction between different variants. Besides, it may not be accurate to evaluate the variants when a deletion or insertion of nucleotide occurs. Many methods take more factors into consideration in order to build a more authentic regulatory model. To name a few, gkm-SVM [30] uses gapped *k*-mers as input features which takes the mismatches in genome into consideration. GERV [44] builds a statistical model assembling ChIP-seq and DNase-seq data to evaluate regulatory variants for TFBSs. Deepsea [45] constructs a deep learning framework to predict the effect of noncoding variants with several kinds of chromatin profiling data. It is somehow difficult to compare these methods to ours due to the different input features. Taking further thought of our method, we can generalize it in estimating the effect of regulatory variants on transcription factor binding sites (TFBSs), eQTLs, histone marks, DNase I–hypersensitive sites (DHSs). For example, we can apply our model to eQTLs analysis with the combination of SNPs and gene expression data and find out which SNPs are more like to be eQTLs. In general, the proposed *kmer*Forest model can still give us an insight when evaluating the impact of regulatory variants according to their changed MDA scores.

## Abbreviations

DHS: DNase I–hypersensitive site
eQTLs: Expression quantitative trait loci
GWAS: Genome wide association study
MDA: Mean decrease accuracy
MSA: Multiple sequence alignment
SNP: Single nucleotide polymorphism
TFBS: Transcription factor binding site
